# 

**Published:** 1819-11

**Authors:** 


### MONTHLY CATALOGUE OF MEDICAL BOOKS.

A Sketch (Analytical) of the History and Cure of Contagious Fever. By Robert Jackson, M.D. 8vo. boards, 8s.

A C. Celsus of Medicine. Translated, with Notes, critical and explanatory, by James Greive, M.D. A new Edition. 12mo. boards, 10s.

Medico-Chirurgical Transactions, Vol. X. Part I. boards, 10s. 6d.

Pathological and Surgical Remarks on Ulcerations of the Genital Organs. By James Evans, Surgeon of his Majesty's 57th Regiment. 8vo. boards, 5s. 6d.

Essays on Hydrocephalus Acutus, or Water in the Brain. By J. Cheyne, M.D. F.R.S.E. &c. &c. 8vo. boards, 7s.

A Catalogue of Books in Anatomy, Medicine, Surgery, Midwifery, Materia Medica, Chemistry, Botany, Veterinary Art, &c. for 1819 and 1820; with a Table of Contents, methodically arranged. By Burgess and Hill. 12mo. 1s. 6d.

Surgical Essays; by Astley Cooper, F.R.S. and Benjamin Travers, F.R.S. Part II. 8vo. boards, 10s. 6d.

